# Inverse Design of Materials by Machine Learning

**DOI:** 10.3390/ma15051811

**Published:** 2022-02-28

**Authors:** Jia Wang, Yingxue Wang, Yanan Chen

**Affiliations:** 1 School of Space and Environment, Beihang University, Beijing 102206, China; wang854@buaa.edu.cn; 2 National Engineering Laboratory for Risk Perception and Prevention, Beijing 100081, China; 3 School of Materials Science and Engineering, Tianjin University, Tianjin 300072, China; yananchen@tju.edu.cn

**Keywords:** inverse design, materials design, machine learning, polymer, photonic, inorganic materials, porous materials

## Abstract

It is safe to say that every invention that has changed the world has depended on materials. At present, the demand for the development of materials and the invention or design of new materials is becoming more and more urgent since peoples’ current production and lifestyle needs must be changed to help mitigate the climate. Structure-property relationships are a vital paradigm in materials science. However, these relationships are often nonlinear, and the pattern is likely to change with length scales and time scales, posing a huge challenge. With the development of physics, statistics, computer science, etc., machine learning offers the opportunity to systematically find new materials. Especially by inverse design based on machine learning, one can make use of the existing knowledge without attempting mathematical inversion of the relevant integrated differential equation of the electronic structure but by using backpropagation to overcome local minimax traps and perform a fast calculation of the gradient information for a target function concerning the design variable to find the optimizations. The methodologies have been applied to various materials including polymers, photonics, inorganic materials, porous materials, 2-D materials, etc. Different types of design problems require different approaches, for which many algorithms and optimization approaches have been demonstrated in different scenarios. In this mini-review, we will not specifically sum up machine learning methodologies, but will provide a more material perspective and summarize some cut-edging studies.

## 1. Introduction

The revolution of materials gave name to different eras of civilization [1,2]. One of the hallmarks of industrialized society is our increasing extravagance in the use of materials. At the same time, the development of other fields enables a deeper understanding of the basis of materials for creating new materials. The enlargement of materials demand, not only in quantity but also in quality, has forced people to explore ways to use existing materials more efficiently, to seek a wide range of new substances as raw materials, to find a way to recycle the waste materials, and to create new materials for specific purposes. The guiding ideology of materials innovation has experienced four paradigms [3]. First, materials innovation relied on empirical trial and error method. Along with the development of mathematics, chemistry, and physics, it came to the second paradigm where people followed scientific laws. The invention of the computer stimulated its application in the scientific field, leading to computational chemistry with computer simulations such as the appearance of Gaussian 70, which can perform ab initio calculations, density functional theory (DFT)-based method, etc. [4,5]. Data-to-knowledge is becoming a new promising solution in materials science as its fourth paradigm by unifying the above three paradigms methodologies in the aspects of theory, experiments, and computer simulation [6]. The powerful fundamental knowledge of materials properties and advanced instruments enables the generation of “big data” and its application of data-driven techniques including data mining, cluster analysis, predictive analytics, genetic programming, visualization of materials dataset, machine learning (ML), business intelligence, learning, and intelligent optimization, etc. [7,8]. These methods have been successfully applied to materials design [9,10,11], chemical synthesis [12,13], and molecular simulations [14,15]. In particular, the emergence of contemporary artificial-intelligence methods and statistic communities [16,17] has provided an astounding new approach to material science and engineering and given birth to the discipline “materials informatics”.

The term “machine learning” was proposed by Samuel in 1959 [18]. ML, with its characteristics of low computational cost and short development cycle, combined with high-quality training data, as well as processing algorithmic methods, allowed for high through-put prediction of experiments of computations [19]. With the possibility of bypassing the solution of complex equations, ML can determine the properties, structure-property relationships, and other data directly through data analysis. For instance, applications in energy, geometry, and the curvature of the potential energy surfaces of molecules have been reported [19]. For materials design, although the multi-objective design requirements and high dimensionality of microstructure space cannot be accomplished by traditional search-based optimization with high efficiency and accuracy in a limited time scale, computational materials design involving ML can successfully provide an accurate way for the nonlinear multi-scale methods to simulate, predict, and select innovative materials [3,20]. Numerous datasets of molecules and materials and their structure-property relationships enable the “learning” and “predicting” of new materials with desirable traits [21]. Furthermore, the use of ML rational resignation is believed to be the most efficient way to replace repetitive lab labor.

Many ML models enable applications in structure determination (phase diagram determination and crystal structure prediction), performance prediction, and fingerprint (descriptor) prediction [22]. Compared to physical-based modeling tools, ML-based density functional theory, molecular dynamics, or the finite element method can offer fast high throughput screening for complex materials analysis, discovery, prediction, and design problems. The ML approaches bring the materials design with a solution to the inherent complexity of searching the vast options [23]. In the materials related ML applications, the principal components are materials descriptors (configuration, topology, fingerprint, etc.) for mapping an input space to an out space, ML algorithms for model training, and optimization process for determining promising candidates [24]. With the development in the above techniques, in recent years ML has been applied to discover new materials from different perspectives, including structure-oriented design (such as the design for polymers, where the chemical composition of the material is predicted from the demanding structure [25]); element-oriented design, where the structure of new compounds is predicted using the composition as input [26]; inverse design, where the target functionality or property is taken as input and the corresponding molecular structure is found out, which generally presented using generative inverse design networks; and drug design, where the structure-activity relationship is accurately predicted using the data from a large number of in vivo functions of small molecules [27]. Currently, the requirement for green chemistry development to mitigate climate change is increasing dramatically. This is a big topic that requires a lot of interdisciplinary, collaborative work. Under such circumstances, materials must revolutionize toward low-carbon or even carbon-free scenarios, which require modified materials or new materials with known functionalities. Moreover, novel materials with unique properties are desperately needed in clean energy technologies [28,29,30,31,32]. The inverse design-based high throughput ML method seems to be a promising area to address materials discovery and materials design. In general, ML-based inverse design uses backpropagation to overcome local minimax traps and performs a quick calculation of the gradient information for a target function concerning the design variable to find the optimizations. In this mini-review, inverse design based on ML and their cut-edging application in several important materials have been reviewed in a limited way.

## 2. Inverse Design

**Figure 1:**
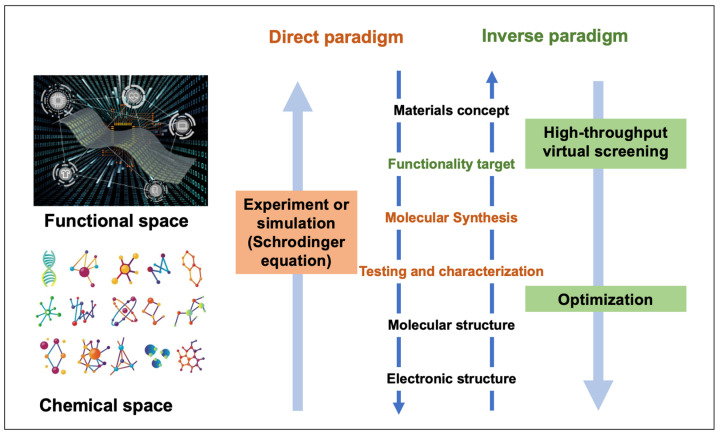
Schematic of the different approaches toward molecular design.

The general molecular design is a nonlinear optimization [33], in which the wave functions, energy eigenvalues, and properties are theoretically explained after the materials designed with unknown molecular structure beforehand by trying experimental optimization [34]. In the so-called direct design (Figure 1), the inputs are the ACS information such as constituent atoms, composition, and structure information database, and the outputs are the properties [35]. In the inverse method of design, one optimizes the properties by varying the wave function coefficients, which then leads to an interpretation of the molecular structure [34]. Inverse design starts from desired properties as “input” and ends in chemical space as “output”, as opposed to the direct approach that leads from the chemical space to the properties [36]. In this way, inverse design (Figure 1) indicates a process that starts with the target functionality, and then the corresponding molecular structure can be mapped to navigate the deliberate chemical application.

**Figure 2:**
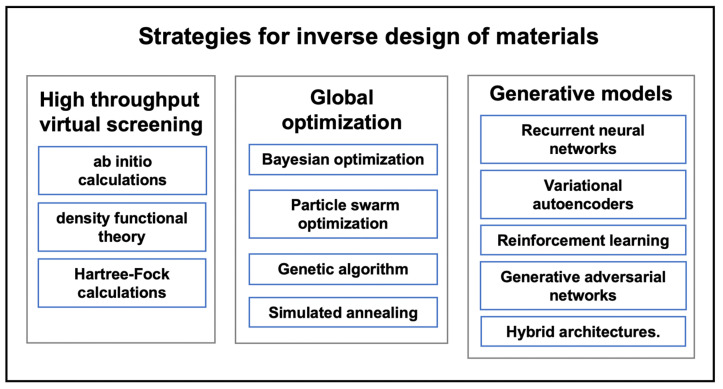
Strategies for inverse design of materials.

Different types of design problems require different approaches. Zunger emphasized three modalities of inverse design: searching artificial superstructures with target functionality, searching the space of chemical compounds for target functionality, and exploring missing compounds for target functionality [35]. The inverse design is usually processed by solving an optimization problem to map a target set of material properties to a subdomain of specific materials, which indicates lengthy calculation in high-dimensional space. To address the above, genetic algorithms (searching the space step by step) and adjoint method (mathematically reversing the equations) are usually used. For example, genetic algorithms or Bayesian framework, etc. can be used through an iterative algorithm [37]. However, inverse design for materials suffers from the extremely vast search space and the requirements for the property evaluation of each sequence [38]. Besides, the inverse design problem is inherently ill-posed or weakly conditioned; when a property or functionality is targeted, there would have a bunch of different types of materials that can satisfy the requirement, which is controversial to “optimize”. To address this problem, methods such as limiting the search space, projecting the search space to a low-dimensional space, using an annealing algorithm, etc. have been applied [39]. To navigate chemical space, three methodologies can be used for materials identification (Figure 2): (1) high-throughput virtual screening; (2) global optimization; and (3) generative models [40,41].

### 2.1. High Throughput Virtual Screening (HTVS)

High throughput virtual screening is a computational investigation of a large set of compounds or materials to assess their qualification for specific requirements. It is best defined by core philosophies as (1) significant timescale; (2) automated techniques; (3) data-driven discovery; and (4) computational funnels [42]. It enables a rather narrow chemical space by defining specific properties, functionalities, building blocks, or bonding rules. The resultant hypothesized candidate from the model usually can be tested by ML-based predictor or high throughput simulations such as molecular dynamics (MD), density functional theory (DFT), finite element method (FEM) etc., which can accelerate the computation process significantly through ML.

**Figure 3:**
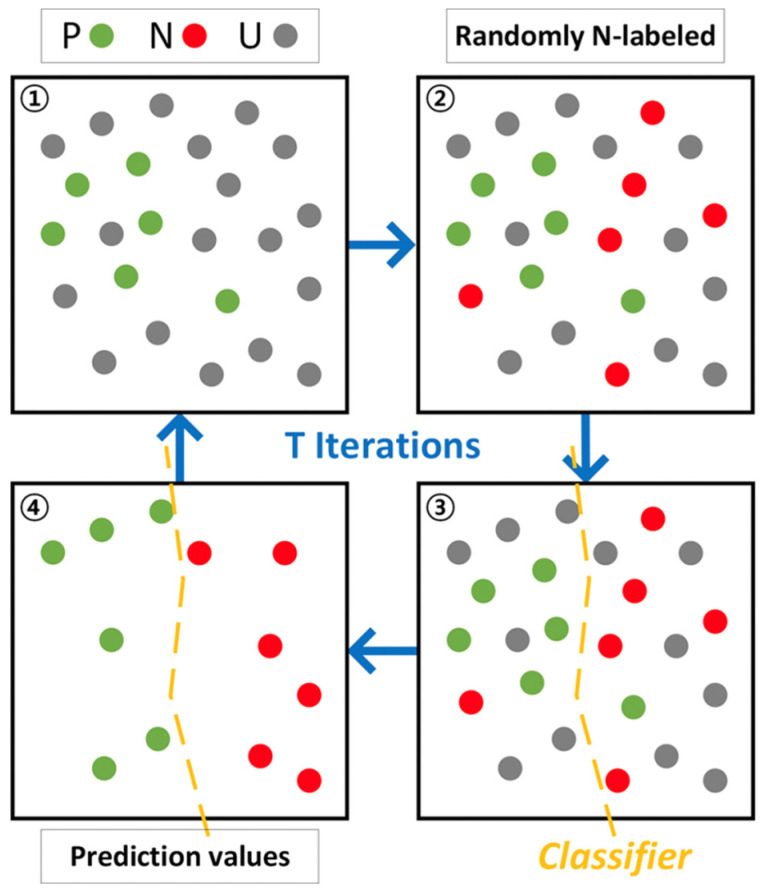
Schematic diagram of positive and unlabeled learning, reprinted with the permission from [43].

For example, Jang et al. [43] proposed a HTVS based on DFT prediction method for inorganic materials synthesis, which is the most important problem in predicting the inorganic materials structures in terms of different functional groups or fragments as in molecules. The MP database for inorganic crystal structures with DFT-calculated properties was used as model training dataset. The graph convolutional neural network (GNN) was implemented as a classifier to the model outputs crystal-likeness scores. Previous developed positive and unlabeled machine learning algorithm combined with GNN-based classifier were used to implement the decision tree. Figure 3 shows the algorithmic of the overall process. P represents a positive data set, which is the organic crystal synthesis data from MP database; U represents unlabeled data set, which is the virtual data from MP; K represent the number of positive data; and T is the number of iterations for bagging. For each iteration, a subsample in U is chosen randomly to be K. After n iterations, twenty percent of P and K are used as classifier and the rest are used as training sets for GNN binary classification model. Then, the classifier predicts that the score will be 1 or 0 based on the similarity to positive-labeled. An average score can be obtained for T times repeating, which represented the synthesizability of a given crystal structure.

Afzal et al. [44] present an HTVS calculation method based on ab initio modeling for the identification of new polyimides with exceptional refractive index values for optical or optoelectronic materials. They defined 29 building blocks as the polyimides’ core structure and made specific moieties structure constraints with respect to certain refractive index by a combination of first principles quantum chemistry calculation and data modeling for the resulting candidate to limit the screening space.

Computational HTVS has been widely used in the discovery strategy in many materials, especially in organic materials, inorganic materials, and organic drugs, each of which has different needs in terms of the number of descriptors, the size of the search space, and the level of approximation. The main problem of HTVS is the size of the library. HTVS need to go through the existing database, but when we design new materials, there is no existing database in our library. However, global optimization (GO) and generative models (GM) are quite different in, that they can capture hidden information from a structure-property-linked database for generating new structures that do not exist in the database.

### 2.2. Global Optimization (GO)

Global optimization is an algorithm to find an optimal solution of the target function and can be applied in the inverse design of various materials, which can help in navigating the chemical space. Bayesian optimization (BO), particle swarm optimization (PSO), genetic algorithm (GA), and stimulated annealing are most seen in materials design. They are potentially useful in multimodal search calculation in inverse problems [45]. For a multi-objective optimization, a function that can normalized the global objectives is needed. For example, we need materials with high *x* properties, low *y* properties, and moderate *z* properties. The optimization of an function *f* (*x*,*y*,*z*) exactly represent the above multi objectives.

BO is systematic approach to find the optimum of function *f* without assumption of any form of *f*. In this way, BO allows acceleration of difficult optimization problems (especially for materials design). In BO, the controllable parameters should be updated to reach the desired objectives. Thus, repeated experiments are needed. For example, Harper et al. [46] used BO with Gaussian processed to obtain eleven different optimal topologies for multi-functional optical materials.

PSO move the optimizers to D-dimensional search space denoted with four vectors: position, velocity, the best position corresponding to the objective function, and the best position found by any of its surroundings. For example, Khadilkar et al. [47] used particle swarm optimization combined with self-consistent-field theory to predict the bulk morphologies in multiblock polymers. In the PSO, the original optimizer agent *i* are described by four vectors: its position $\vec{x_{i}}=\left(x_{i1},x_{i2},\ldots ,x_{iD}\right)$, its velocity $\vec{v_{i}}=\left(v_{i1},v_{i2},\ldots ,v_{iD}\right)$, the post position corresponding to the objective function $\vec{p_{i}}=\left(p_{i1},p_{i2},\ldots ,p_{iD}\right)$, and the best position found by its neighbors $\vec{n_{i}}=(n_{i1},n_{i2},\ldots \;n_{iD})$. Thus, agent *i* in *d* dimension can be described as:

$$\begin{array}{c} v_{id}^{n+1}=v_{id}^{n}+\chi c_{0}\phi_{0}\left(p_{id}-x_{id}\right)+\chi c_{1}\phi_{1}\left(n_{id}-x_{id}\right)-\left(1-\chi \right)v_{id}\\ x_{id}^{n+1}=x_{id}^{n}+v_{id} \end{array}$$

where *ϕ*_0_ and *ϕ*_1_ are independent, uniformly distributed random variables in the interval [0, 1] generated at every update, and *c*_0_ and *c*_1_ are acceleration coefficients. The parameter *χ* ∈ [0, 1] is known as the constriction factor. After PSO search, the fitness is certified by self-consistent-field theory for the target phase and candidate phases. They found the procedure is robust in polymer design using bulk information as a describer and can be broadened to targeting properties directly (for example, photonic bandgap).

**Figure 4:**
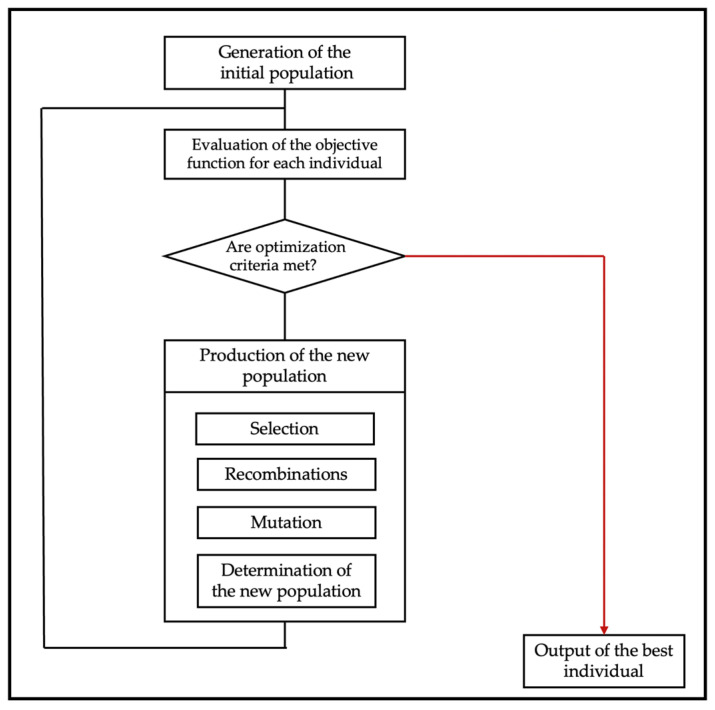
Structure of a simple genetic algorithm.

GA, similar to PSO, uses a population of points or variables to propose potential solutions. It is inspired by the natural biological evolutionary process with steps of crossover, mutation, selection, and passing on the selected genes to the next generation. The structure of a simple GA is shown in Figure 4. GA is suitable for exploring large search spaces and thus can be effectively used for in materials inverse design, especially in the molecular search space. For example, Lee et al. [48] introduced a novel two phase GA method as constrained optimization for molecular inverse design while constraining the molecular structure. Self-referencing embedded strings and graph are used as descriptors for mutation and crossover, respectively, which generate valid molecular candidates and allow new molecules to be generated by random editing, but with appropriate target properties and limited structural information and without previous experience rules. In the new strategy, they first construct a population that is always valid for the existing dataset and a second stage was built to select suitable molecular descriptors to ensure the validity of the generated molecules. They showed that the model can preserve the molecular core and optimize target protein properties across generations through cannabidiol molecular optimization.

### 2.3. Generative Models (GM)

GM is unsupervised learning that encodes the high-dimensional materials chemical space into the continuous vector space (or latent space)with lower dimensionality, and generates new data using knowledge embedded in the vector space [36]. Thus, it is able to synthesize novel, high dimensional data samples. Several GM approaches have been used for inverse design of materials, and to the best of our knowledge, the most commonly used for various materials are recurrent neural networks (RNNs), variational autoencoders (VAEs), reinforcement learning (RL), generative adversarial networks (GANs), and hybrid architectures [49].

**Figure 5:**
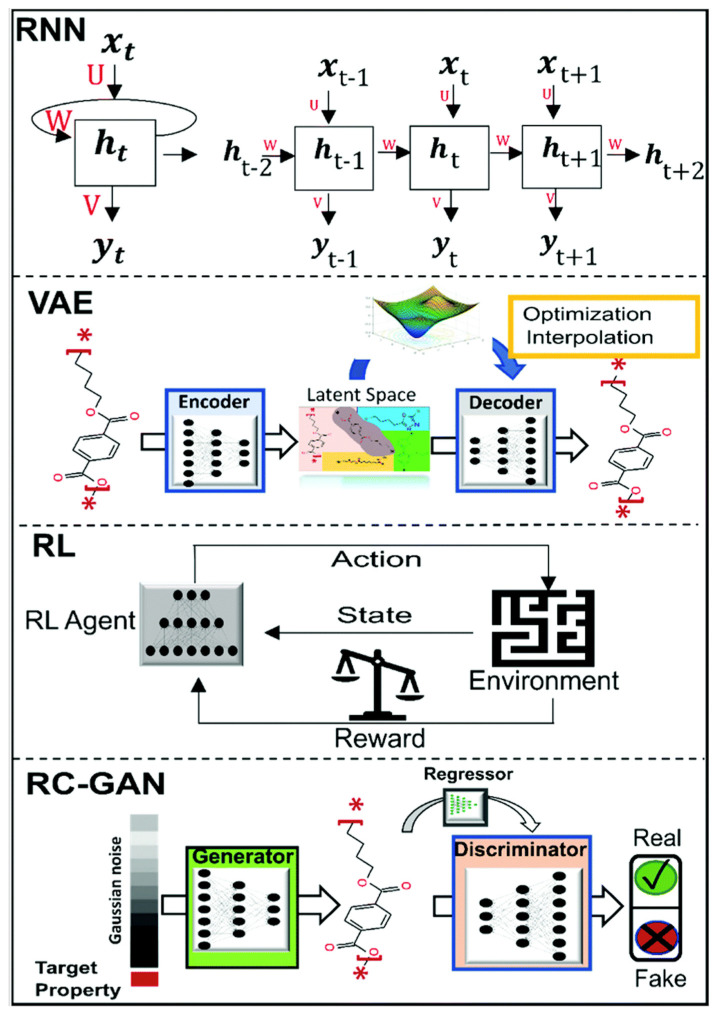
DL-based algorithms for GMs. (Reprinted with the permission from [41]).

RNNs can generate sequences from incrementally one step at a time and predicting what comes next based on the current and past information. RNNs do not need static input data, as shown in Figure 5. Current input vector $X_{\left(t\right)}$ and the past knowledge $h_{\left(t-1\right)}$ at time step t are the input vector, allowing RNNs to generate sequential data based on the learning information of the last iteration. For example, Kim et al. [50] implemented a hybrid deep encoder-decoder architecture method for discovery of organic molecules, which a deep neural network (DNN) was adopted as the encoder to identified the relationship between structural features and their material properties and RNNs were adopted and the decoder to reconstructed the recognizable molecular structures from the hidden relationship.

AE generally includes an encoder to encode molecules to a continuous vector in a lower dimension and decoder maps for the vector back to obtain the original representation (as shown in Figure 5). The encoder–decoder architecture of VAEs enable better generalizability by constraining the encoder network with a probability distribution [36]. In the inverse design of materials, with the advantages of combining neural networks and probability models, VAE enables the processing of large and complicated datasets. Moreover, continuous representation launches the gradient-based optimization models to decode arbitrary vectors and interpolate structures. For example, Ma et al. [51] described a VAE structure to metamaterial design problem. They defined three variables as input variable *x* (geometric pattern of metamaterial structure), output variable *y* (three distinct reflection spectra), and latent variable *z* (compressed code of the design). A probabilistic relationship between the above three variables was established by a VAEs model. Each probabilistic relationship represents different functionalities of the metamaterials. Their models showed the ability to simultaneously solve the forward and inverse problem, which is predominant compared to GAN, which requires a pre-trained simulator to guarantee the inverse process.

RL considers the generator as agent and studies how an agent interacts with an environment or task to maximize some notion of reward (properties), as shown in Figure 5. RL is a subfield of AI, which is used to solve dynamic decision problems. For example, Popova et al. [52] devised a novel computational strategy based on deep RL for generating chemical compounds with desired physical, chemical, and/or bioactivity properties de novo. They implement two deep neural networks (a generative model and a predictive model) in deep RL framework, which the generative model is used to generate chemically feasible molecules and the predictive model estimates the agent’s behavior by assigning a numerical reward (or penalty) to every generated molecule. The generative model is trained to maximize the reward.

GAN consists of a generator and a discriminator, which are trained simultaneously with conflicting objectives. The generator takes in a noise vector and outputs an image, while the discriminator takes in an image and outputs a prediction about whether the image is a sample from generator. Competition of the generator and the discriminator improves both networks while generator is trained to maximize the probability that discriminator makes a mistake, and discriminator is trained to minimize that probability. For example, Geng et al. [47] adopt a GAN in network model for inverse design of metasurfaces for dielectric materials. In the work, structure-property relationships and generated optical spectrum are simulated by GAN, and rational design prediction is made. The simulator is a pretrained fixed-weight model that takes the generated patterns as input and approximates their transmission spectra without the use of electromagnetic simulation. The distance of user-defined geometric data and the patterns from the generator was minimized by backpropagation training.

## 3. Application in Materials Design

### 3.1. Polymers

Polymeric materials are widely used in various aspects of everyday life and technological development, such as actuators, agriculture, aviation, biomedicine, biosensing devices, catalysts, chemotherapy, chitosan, electronics, fuel cell, furniture, membranes, packaging, textile, etc. due to their attractive physical, chemical and electrical properties [53]. The demand for polymers with better performance and lower carbon footprint is driving the design of new polymeric materials. Polymer dynamics and chemo-functionality determine the polymer properties, while the inverse design provides an approach to design polymers based on the desired attributes and a ML approach can make rapid predictions due to the rapid inference rate of ML-based predictive modeling [39]. However, due to the chemical, topological, and morphological complexity of polymers and various synthesis information, research is scarce and mostly computationally expensive; the related field is still in its infancy. The inverse design of polymers in both ML and deep learning methods has been well-reviewed by Sattari et al. [41] and ML for polymer design has been well summarized by Kumar et al. [54]. The data-driven algorithms for inverse design of polymers have two paths to follow in general: high throughput virtual screening and smart search algorithms [36]. These have been well-reviewed by Sattari et al. [41]. Here in this paper, some highlight inverse designs of the polymer by ML will be emphasized.

**Figure 6:**
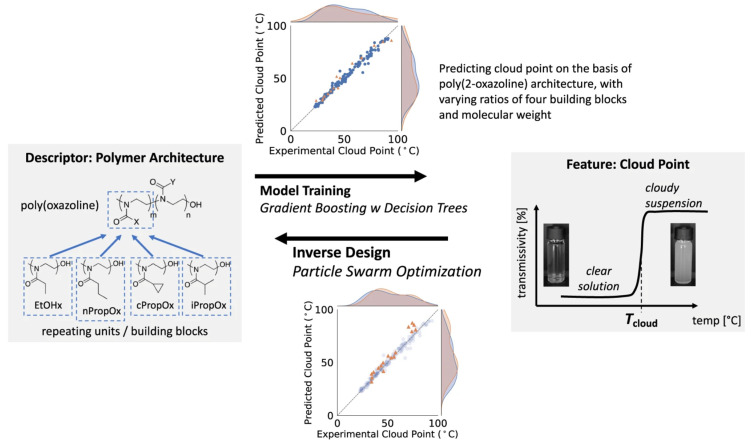
Study framework of polymer cloud-point engineering via machine learning inverse design, Reprinted with the permission from [55].

Phase behavior is a feasible target property for polymer inverse design, it is strongly influenced by polymer structures, polymer-polymers interactions, solution, etc. Based on target-phase properties, such as cloud point, polymer structure information including size, topology, composition, functionality can be derived by ML. Kumar et al. [55] developed an ML method based on particle swarm optimization for tuning of poly(2-oxazoline) cloud point with high accuracy (Figure 6). Four building blocks were identified as descriptor for polymer architecture, by which the machine learning model was trained to predict the cloud point. The model, consisting of a trained algorithm and PSO, was demonstrated by predicting 17 polymer structures with desired cloud point. Incidentally, PSO is often used in the polymer inverse design. It is a bioinspired search technique t suitable for complex systems with divergent distribution and solves the problem without centralized control in a specific individual [56]. Khadilkar et al. [57] used particle swarm optimization to predict the bulk morphologies in multiblock polymers, using separate self-consistent-field theory to ensure accurate estimation of the equilibrium structure. Their methodology was demonstrated suitable for single multiblock polymers as well as blend systems and even more block copolymers. Hiraide et al. [58] predicted the phase separation structure of polymer alloy from specific properties. They trained the framework by the convolutional neural network from previous analysis to predict the phase separation structure of a polymer alloy, subsequently applied a hybrid model consisting of a generative adversarial network and convolutional neural network. The framework they built was demonstrated as a low-cost method.

**Figure 7:**
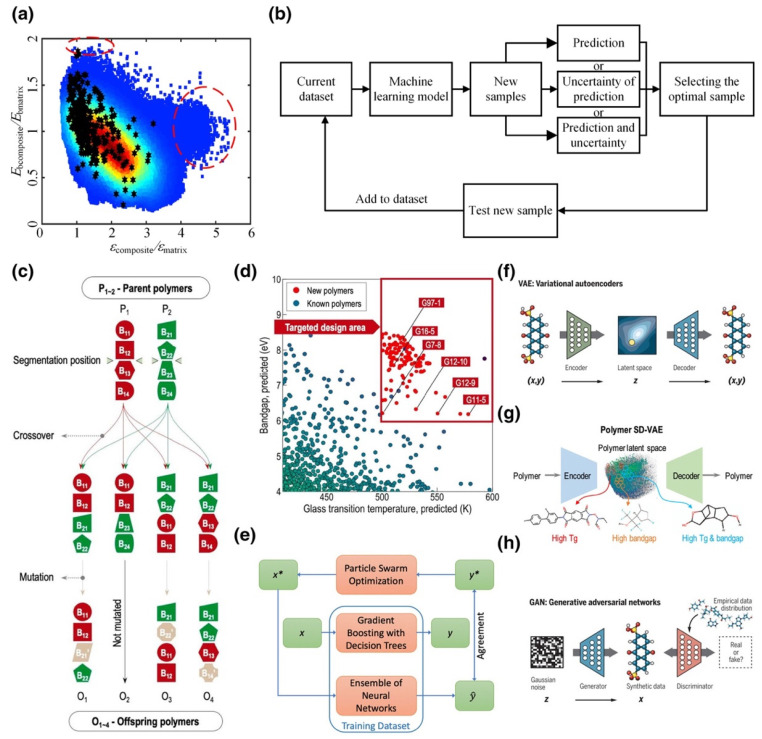
Inverse design methods for polymer-based dielectrics. (**a**) Enumeration method. (**b**) Active learning algorithm. (**c**,**d**) Genetic algorithm method used to design polymers with high glass transition temperature and large bandgap. (**e**) Inverse design method based on particle swarm optimization. x* refers to polymer design and (y*) refers to desired cloud-point. (**f**) Variational autoencoder (VAE). (**g**) VAE used to discover polymers with high Tg and bandgap. (**h**) Generative adversarial networks. Reprinted with the permission from [60].

Polymer dielectrics are essential properties, especially when used in capacitive energy storage, organic photovoltaics. Diverse spectrum information and high data availability provide sufficient training models for ML techniques for polymer design (Figure 7). However, the vastness of polymer chemical and structural space could conceal some key opportunities. There are mainly two distinct steps for the above scenario: fingerprinting polymers into numerical representations and establishing a mapping between the numbers and target property [59]. Several ML algorithms are commonly used in these calculations, such as linear regression, GPR, ANN, RF, deep neural network, etc. [60] Mannodi-Kanakkithodi et al. [61] addressed the polymer dielectric design by ML-based genome approach for optimization of polymer constituent blocks, where they fingerprinted polymers into easily attainable numerical representations in prior. Their method accelerates the discovery of on-demand polymers with desired dielectric constant. Wu et al. [62] processed an algorithm based on inference and sampling with sequential Monte Carlo to target dielectric constant and bandgap. Gurnani et al. [63] proposed a graph-to-graph translation based novel ML algorithm called polyG2G to inverse design the polymer dielectrics. They trained the system with a high range of performance polymers and analyzed the subtle chemical differences between them. The difference continuously became an index from high throughput screening. Thousands of potential targets in an intractable search space with desired glass-transition temperatures, bandgap, and electron injection barriers have been found by the novel algorithm.

The self-assembly of block copolymers, which have robust application in medicine, can be designed through tuning the phase behavior to achieve exotic structures [64]. However, to achieve the inverse design of copolymers, expert knowledge and much time is needed for the selection of order parameters. Moreover, the results of simulation have nowhere to confirm as comprehensive. Patra et al. [38] used a Monte Carlo tree search to minimize the total number of evaluations in a given design cycle to copolymer compatibilizer design, which is inspired by AI gaming algorithms. They established a framework that combined the algorithm with molecular dynamics simulations, then applied it to specific polymer chain lengths to confined overall search space. The framework can also be extended to several proteins.

### 3.2. Photonic

**Figure 8:**
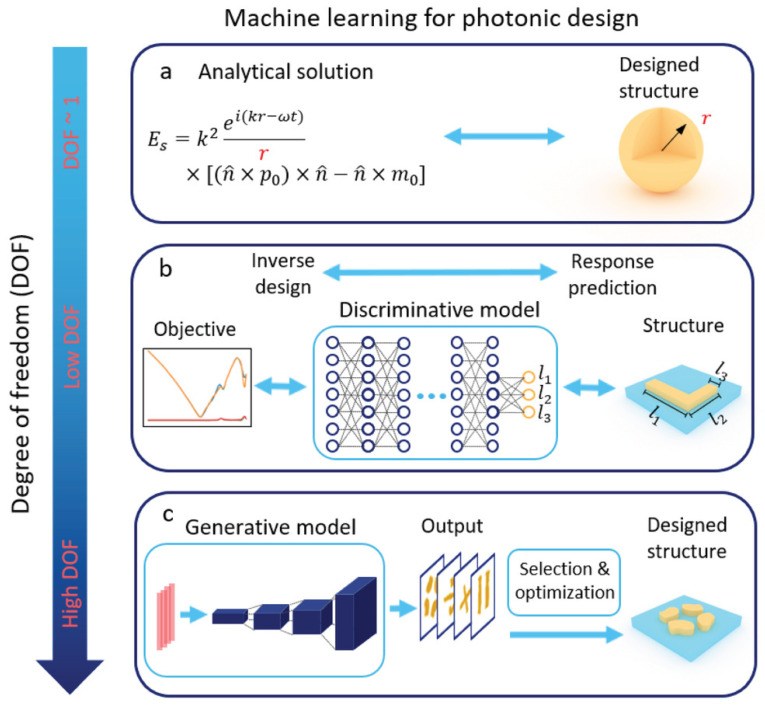
Methodologies of photonic design through machine learning at different degrees of freedoms (DOFs), reprinted with the permission from [64].

Integrated photonics including materials and devices are widely applied in optical communication, biomedicine, biomedical, sensing technologies, etc. [65]. They can be accurately manipulated by changing the structure and degrees of freedom (DOF). To achieve target properties in transmittance, polarization, chirality, frequency, etc., researchers have made many efforts in the design of microscopic structures of photonics. Although it is quite understandable that the photonics performance from the knowledge of photonics structures should be predicted, inverse design of on-demand photonics is another story altogether and understandably represents a much more recent development [66]. The background and development history of inverse design in nanophotonic has been well-reviewed by Molesky et al. [67]. The methodologies of photonic design through machine learning at different degrees of freedoms are shown in Figure 8. When DOF of photonics structure is low, either a simple analytical solution or parametric sweeping can be used for the optimization. However, the simple methods suffer from low reliability. The solution space becomes larger as the DOF increases, and discriminative model can be used for the structure-property relationship. However, this approach often fails to find a particular optimal design parameters since multiple structures will produce the same response accordingly. If DOF continue to increase to thousands and more, a generative model can be used to reduce the dimensionality of the chemical space, a good optimization algorithm can be applied to locate an optimization.

The photonic inverse design is typically solved by local optimization as other physical design problems [68]. Traditional optimizations such as adjoint methods, GA, and PSO, have been applied to photonics design but with expensive computation and local minimum problems since it requires the same large amounts of simulations for each design, while ML only needs limited training for neural networks due to its ability to identify hidden correlations in the large data sets during the training phase. More importantly, once the neural networks are trained for a complex system problem, it can approximate the same computation in orders of magnitude less time owing to the ability to retrieve knowledge allows the simulations to be invested in the design tool and can be applied to each design without costly computations [69]. Besides, some approaches that available to ML models can enhance the likelihood of achieving the global minimum in the optimization problems. Thus, ML as a stand-alone technique can help the inverse design of photonics and on the other hand, photonics provides a place to solve ML problems [65]. However, inverse designs have issues such as low training efficiency when dealing with inconsistent data, and inverse problems in photonic design often generate scattering problems. Therefore, the training process and optimization methodology are important. Qu [70] et al. established an optical neural network framework based on optical scattering units by introducing the “kernel matrix”. Micrometer-level footprint allows an accelerated process for deep learning. Their framework demonstrated 97.1% accuracy but with an inefficient training process. They suggested that in situ training on the integrated photonics probably can help the framework further decrease their footprints and not sacrifice efficiency and functionality at the same time.

Topology optimization is a good computational tool that can be used for the systematic design of photonic crystals, waveguides, resonators, filters, and plasmonic, and the related logic and mathematics has been well-reviewed by Jensen et al. [71]. This is owing to the gradient descent nature of topology optimization, such as steepest descent and conjugate gradient, which can provide a reduction of constraints for an objective function [72]. Due to materials’ complex optical response and geometrical structure, the photonics design with tuning targeted topology remains a challenge. Long et al. [73] proposed an ML approach to design optical structures with the target topological states in a one-dimensional dielectric photonic crystal system. In the system, the Zak phase was descripted as state vectors and label vectors, referring to the geomatical information and reflection phase properties respectively. The neural network was trained by a tandem pipeline to establish the inverse design model. The optical structure can be acquired by applying targeted topological properties. Pilozzi et al. [74] employ a supervised ML regression to design photonic topological insulators. Aubry–Andre–Harper band structure models are used for neural networking training and a twist based on a reverse validation between the inverse problem neural network and the direct problem neural network has been introduced to ensure the only solution can be found. The method can be extensively applied to other physical systems in topological science, such as polaritonic, quantum technologies, and ultra-cold atoms, as well as 2D and 3D topological systems, quantum sources, and simulations. With the development of advanced deep learning algorithms, generative adversarial networks and autoencoder extended the possibility to joint with topology optimization to perform optimization in a complex topological system. Jiang et al. [75] demonstrated generative adversarial neural networks are effective for nanoantenna design optimization and can generate high-performance metasurfaces when coupling with topology optimization. Liu et al. [76] propose an encoding method for binary images that represent the topology of photonic structures for data generation and dimensionality reduction. The method was demonstrated and proved the ability to provide a way to generate global optimization results within limited solution space as well as enhance the accuracy of the network. Kudyshev et al. [77] used an adversarial autoencoder coupled with a metaheuristic optimization framework to assist global optimization of photonic devices with complex topologies.

### 3.3. Inorganic Solid-State Functional Materials

The discovery of novel inorganic functional materials is the core of many technologies’ development such as solid electrolytes for lithium-ion batteries, robust membrane for capturing carbon dioxide, halide perovskites for perovskite solar cells, etc.

**Figure 9:**
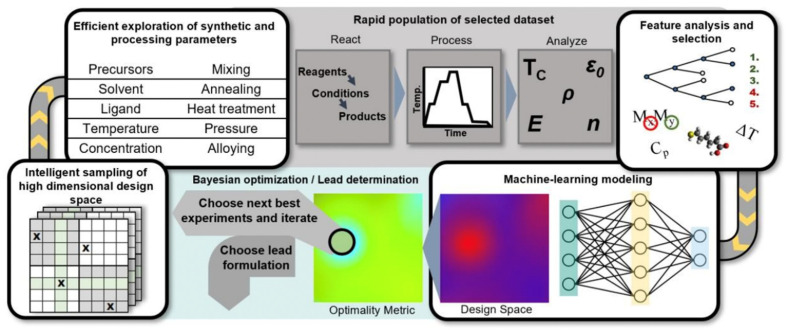
Schematic depiction of an example of a machine-learning workflow for the iterative exploration and exploitation of a synthetic design space for inorganic materials, reprinted with the permission from [78].

For inorganic substances, molecular simulations and first-principles methods are commonly used methodologies, but they are computationally expensive for large chemical space screening. Recently, HTVS based on density functional theory (DFT) calculations have become a rather popular topic, which allows the discovery of crystals with targeted functional properties. However, the above methods focus on screening based on the existing dataset, which means that regressing the crystal or moieties representations can meet the required properties, whereas ML based on global optimization allows inverse design/discovery of new crystals with on-demand properties. This approach generally requires a structural pool of chemical compositions and their corresponding properties. Moreover, probabilistic generative models to existing materials to a continuous latent space can also lead to inverse materials design through mapping the latent space to materials properties. Indeed, there is a vital challenge in inorganic materials design. For example, a significant number of after screening hypothetical crystals are not observed in experiments, a thermodynamic model of crystals is simplified in prior which could lead to the inaccurate descriptor. Another challenge in inorganic materials synthesis design by ML is the high dimensionality of the problems. Synthesis is generally involved in many different parameters including the reactants parameters and synthesis environmental parameters, where *n* synthesis variables create an *n* dimension exploration space. Figure 9 shows a typical schematic depiction of ML workflow for inorganic materials design [78].

Many exciting developments have been well-established by Noh et al. [79]. Chen et al. [40] has reviewed the generative models for inverse design of inorganic solid material. Zunger [35] discussed the inverse design of solid-state materials with target functionalities very comprehensively. Only limited works will be mentioned in this mini-review.

HTVS, GO, GM, GAN, and support vector machine regression (SVM) are usually used for inorganic materials inverse design. Kim et al. [80] proposed a generative framework using evolutionary algorithms and quasi-random searching. The framework is inversion-free with a relative low memory requirement on the unit cell. Fractional atomic coordinates are used as crystal representations to build the crystal structures. Atomic coordinates and cell parameters are projected to the ML field by image classification and segmentation, which are used as a set of points and vectors with 3D coordinates. They demonstrated the effectiveness of the framework by asking for photoanode properties for high-throughput virtual screening with the generation of Mg–Mn–O ternary materials. Dan et al. [81] proposed the first GAN model to efficiently sample the inorganic material design space by generating hypothetical inorganic materials. The Open Quantum Materials Database, Materials Project, and ICSD databases have been used for model training of chemical compositional rules. Their application experiments showed that 2 million targeted materials were obtained with as high as 92.53% materials novelty. Rosales et al. [82] describe a HTVS to the inverse design of enantioselective catalyst candidates, substrate and ligand libraries or asymmetric catalysis was screening within hours. SVM was then used to generate a visual map of the space. Braham et al. [78] studied CsPbBr3 perovskite nanocrystal growth by SVM to initially separate regions of the design space that yield quantum-consolidated nanoplatelets from regions that yield bulk particles. Further predictions can also be made by the model, and it provides a perspective on the influence of molecular ligands on the dimensions of nanocrystals.

### 3.4. Porous Materials

Porous materials are widely used in catalysis, separations, sensors, electronics, architecture, biomedical, and electronics [83,84]. A rational design for porous materials with regular, accessible cages and tunnels is now being demanded. Neural networks based on ML can be applied to materials’ compositions, bandgap energy, formation energy, and gas adsorption uptakes, which is an appropriate method for porous materials such as zeolites, metal-organic framework, etc. However, it is challenging work due to the complex chemistry of these porous materials. For example, they contain various unit cells and unclear lattice parameters. Kim et al. [85] proposed an artificial to generate pure silica zeolite structures, which a generative adversarial network are used for training. Yao et al. [86] applied generative models for nano-porous l neural network crystalline reticular materials (metal-organic framework) inverse design. They demonstrated that autoencoder is a promising optimization method for metal-organic framework related predication when trained with multiple top adsorbent candidates identified for superior gas separation. Wan et al. [87] reported an ML-based inverse design of porous graphene. In their research, they build up a relationship between hole distribution and thermal conductivity reduction in monolayer graphene by machine learning method. This is then used for backpropagation to generate porous graphene with low thermal conductivity.

### 3.5. Other Materials

There are many other materials have been designed through inverse design approach based on the ML method. Thermoelectric materials represent highly efficient solid state energy conversion and play a role in both primary power generation and energy conservation. The design of it drawing many attentions and the ML-based method can provide a rational design method. The machine learning approaches for thermoelectric materials have been well reviewed by Wang et al. [88] and Gomez et al. [89]. Here, some other materials related research are lists in Table 1 as below.

## 4. Challenges and Opportunities

Inverse design navigates to material innovation by taking the targeted functionality or property as input to obtain an output of structural material information. It is a promising strategy to accelerate the discovery of materials and shorten the time for technology development, whose direct design requires much more time on trial-error experiments. Traditionally, inverse problem are generally solved by mathematically inverting the Schrödinger equation. However, it is usually not practical to find the inversion of this equation due to mathematical restrictions, the complex physical system of the materials design, and a scalable approach that leverages the talent and efforts of the entire materials community. Data driven techniques provide a different way for inverse problem, which requires no mathematical inversion of any equation but to manipulate a large set of direct approach calculation to find the relationship between the properties/functionalities and molecule structures. ML as a component tool for data driven inverse design is rapidly developing. The ML-based approaches can quickly map between the fingerprinted input and the target properties by using backpropagation to overcome local minimax traps and performs a quick calculation of the gradient information for a target function with respect to the design variable to find the optimizations. It can produce logical framing of chemical space, better exploration of chemical space within required regions, and optimization methods. ML-based approaches are highly available for multi-objective design requirements and the high dimensionality of microstructure space, which cannot be achieved by traditional statistical methodologies. However, there are many challenges. One of the most vital challenges in inverse design, or rather in all data-driven materials design, is the close and iterative interaction between theories and experiments. How to realize the predictions and how to produce predicted materials must be considered. Building an invertible and invariant generative model is quite a challenge due to the lack of an explicit approach for the permutation and combination of different conditions without exploring the entire design space. Another important challenge is to develop an experimental feedback loop which can enhance the reliability of the decisions from the artificial intelligent. As seen, the integration of ML as a new pillar of knowledge in materials will simulate a related application throne, while the application scenario also provides a place to solve ML problems, such as photonics, different catalysis, ultrafast nanomaterials, 2-D materials, etc. [23,24,95,96,97,98].

## Figures and Tables

**Table 1:** Other advanced materials inverse design by machine learning.

Materials/Molecules	Methodology	Target	Reference
Acoustic metamaterials	Gauss-Bayesian model	Specific functionalities	[90]
Photovoltaic	GA using developed MATLAB code	Voltage-current relation of the PV module.	[91]
Organic molecules	RNN	Relation between molecular structures and their material properties	[49]
Self-assembling materials	statistical mechanics based approach	Complex microstructures	[92]
Soft membranes	Neural network	3D shapes starting from 2D planar composite membranes	[93]
Thin-film materials	Neural networks	Relationships between the metamaterial structure and corresponding ellipsometric and reflectance/transmittance spectra	[94]
Colloidal crystals	Alchemical Monte Carlo simulation	Geometric shape structure	[52]

## References

[1] Sass S.L. (1998). The Substance of Civilization: Materials and Human History from the Stone Age to the Age of Silicon.

[2] Headrick D.R. (2000). When Information Came of Age: Technologies of Knowledge in the Age of Reason and Revolution, 1700–1850.

[3] Agrawal A., Choudhary A. (2016). Perspective: Materials informatics and big data: Realization of the “fourth paradigm” of science in materials science. APL Mater..

[4] Pople J.A. (1999). Quantum Chemical Models (Nobel Lecture). Angew. Chem. Int. Ed..

[5] Garrity K.F., Bennett J.W., Rabe K.M., Vanderbilt D. (2014). Pseudopotentials for high-throughput DFT calculations. Comput. Mater. Sci..

[6] Hobart M.E., Schiffman Z.S. (2000). Information Ages: Literacy, Numeracy, and the Computer Revolution.

[7] Mosavi A., Vaezipour A. (2012). Reactive Search Optimization; Application to Multiobjective Optimization Problems. Appl. Math..

[8] Rajan K. (2013). Informatics for Materials Science and Engineering: Data-Driven Discovery for Accelerated Experimentation and Application.

[9] Mosavi A., Rabczuk T., Varkonyi-Koczy A.R. (2018). Reviewing the novel machine learning tools for materials design. Recent Advances in Technology Research and Education.

[10] Goh G.B., Hodas N.O., Vishnu A. (2017). Deep learning for computational chemistry. J. Comput. Chem..

[11] Dam H.C., Pham T.L., Ho T.B., Nguyen A.T., Nguyen V.C. (2014). Data mining for materials design: A computational study of single molecule magnet. J. Chem. Phys..

[12] Coley C.W., Green W.H., Jensen K.F. (2018). Machine Learning in Computer-Aided Synthesis Planning. Acc. Chem. Res..

[13] Kim E., Huang K., Jegelka S., Olivetti E. (2017). Virtual screening of inorganic materials synthesis parameters with deep learning. npj Comput. Mater..

[14] Mardt A., Pasquali L., Wu H., Noe F. (2018). VAMPnets for deep learning of molecular kinetics. Nat. Commun..

[15] Chen C., Lu Z., Ciucci F. (2017). Data mining of molecular dynamics data reveals Li diffusion characteristics in garnet Li_7_La_3_Zr_2_O_12_. Sci. Rep..

[16] Gopnik A. (2017). Making AI More Human. Sci. Am..

[17] Jordan M.I., Mitchell T.M. (2015). Machine learning: Trends, perspectives, and prospects. Science.

[18] Provost F., Kohavi R. (1998). Glossary of terms. J. Mach. Learn..

[19] Wei J., Chu X., Sun X.Y., Xu K., Deng H.X., Chen J., Wei Z., Lei M. (2019). Machine learning in materials science. InfoMat.

[20] Fischer C.C., Tibbetts K.J., Morgan D., Ceder G. (2006). Predicting crystal structure by merging data mining with quantum mechanics. Nat. Mater..

[21] Takahashi K., Tanaka Y. (2016). Material synthesis and design from first principle calculations and machine learning. Comput. Mater. Sci..

[22] Liu Y., Niu C., Wang Z., Gan Y., Zhu Y., Sun S., Shen T. (2020). Machine learning in materials genome initiative: A review. J. Mater. Sci. Technol..

[23] Moosavi S.M., Jablonka K.M., Smit B. (2020). The Role of Machine Learning in the Understanding and Design of Materials. J. Am. Chem. Soc..

[24] Ward L., Agrawal A., Choudhary A., Wolverton C. (2016). A general-purpose machine learning framework for predicting properties of inorganic materials. npj Comput. Mater..

[25] Kim C., Batra R., Chen L., Tran H., Ramprasad R. (2021). Polymer design using genetic algorithm and machine learning. Comput. Mater. Sci..

[26] Seko A., Hayashi H., Nakayama K., Takahashi A., Tanaka I. (2017). Representation of compounds for machine-learning prediction of physical properties. Phys. Rev. B.

[27] Burbidge R., Trotter M., Buxton B., Holden S. (2001). Drug design by machine learning: Support vector machines for pharmaceutical data analysis. Comput. Chem..

[28] Linares N., Silvestre-Albero A.M., Serrano E., Silvestre-Albero J., García-Martínez J. (2014). Mesoporous materials for clean energy technologies. Chem. Soc. Rev..

[29] Liu S., Shen Y., Zhang Y., Cui B., Xi S., Zhang J., Xu L., Zhu S., Chen Y., Deng Y. (2021). Extreme Environmental Thermal Shock Induced Dislocation-Rich Pt Nanoparticles Boosting Hydrogen Evolution Reaction. Adv. Mater..

[30] Liu C., Zhou W., Zhang J., Chen Z., Liu S., Zhang Y., Yang J., Xu L., Hu W., Chen Y. (2020). Air-Assisted Transient Synthesis of Metastable Nickel Oxide Boosting Alkaline Fuel Oxidation Reaction. Adv. Energy Mater..

[31] Liu S., Hu Z., Wu Y., Zhang J., Zhang Y., Cui B., Liu C., Hu S., Zhao N., Han X. (2020). Dislocation-Strained IrNi Alloy Nanoparticles Driven by Thermal Shock for the Hydrogen Evolution Reaction. Adv. Mater..

[32] Wu H., Lu Q., Zhang J., Wang J., Han X., Zhao N., Hu W., Li J., Chen Y., Deng Y. (2020). Thermal Shock-Activated Spontaneous Growing of Nanosheets for Overall Water Splitting. Nanomicro. Lett..

[33] Press W.H., Flannery B.P., Teukolsky S.A., Vettering W.T. (2002). Numerical Recipes in C: The Art of Scientific Computing.

[34] Kuhn C., Beratan D.N. (1996). Inverse Strategies for Molecular Design. J. Phys. Chem..

[35] Zunger A. (2018). Inverse design in search of materials with target functionalities. Nat. Rev. Chem..

[36] Sanchez-Lengeling B., Aspuru-Guzik A. (2018). Inverse molecular design using machine learning: Generative models for matter engineering. Science.

[37] Peurifoy J., Shen Y., Jing L., Yang Y., Cano-Renteria F., DeLacy B.G., Joannopoulos J.D., Tegmark M., Soljacic M. (2018). Nanophotonic particle simulation and inverse design using artificial neural networks. Sci. Adv..

[38] Patra T.K., Loeffler T.D., Sankaranarayanan S. (2020). Accelerating copolymer inverse design using monte carlo tree search. Nanoscale.

[39] Wu S., Yamada H., Hayashi Y., Zamengo M., Yoshida R. (2020). Potentials and challenges of polymer informatics: Exploiting machine learning for polymer design. arXiv.

[40] Chen L., Zhang W., Nie Z., Li S., Pan F. (2021). Generative models for inverse design of inorganic solid materials. J. Mater. Inform..

[41] Sattari K., Xie Y., Lin J. (2021). Data-driven algorithms for inverse design of polymers. Soft Matter.

[42] Pyzer-Knapp E.O., Suh C., Gómez-Bombarelli R., Aguilera-Iparraguirre J., Aspuru-Guzik A. (2015). What is high-throughput virtual screening? A perspective from organic materials discovery. Annu. Rev. Mater. Res..

[43] Jang J., Gu G.H., Noh J., Kim J., Jung Y. (2020). Structure-Based Synthesizability Prediction of Crystals Using Partially Supervised Learning. J. Am. Chem. Soc..

[44] Afzal M.A.F., Haghighatlari M., Ganesh S.P., Cheng C., Hachmann J. (2019). Accelerated Discovery of High-Refractive-Index Polyimides via First-Principles Molecular Modeling, Virtual High-Throughput Screening, and Data Mining. J. Phys. Chem. C.

[45] Scales J.A., Smith M.L., Fischer T.L. (1992). Global optimization methods for multimodal inverse problems. J. Comput. Phys..

[46] Harper E., Mills M. (2020). Bayesian Optimization of Neural Networks for the Inverse Design of All-Dielectric Metasurfaces.

[47] Geng Y., van Anders G., Glotzer S.C. (2018). Predicting colloidal crystals from shapes via inverse design and machine learning. arXiv.

[48] Lee Y., Choi G., Yoon M., Kim C. (2021). Genetic Algorithm for Constrained Molecular Inverse Design. arXiv.

[49] Odena A. (2016). Semi-supervised learning with generative adversarial networks. arXiv.

[50] Kim K., Kang S., Yoo J., Kwon Y., Nam Y., Lee D., Kim I., Choi Y.-S., Jung Y., Kim S. (2018). Deep-learning-based inverse design model for intelligent discovery of organic molecules. npj Comput. Mater..

[51] Ma W., Cheng F., Xu Y., Wen Q., Liu Y. (2019). Probabilistic Representation and Inverse Design of Metamaterials Based on a Deep Generative Model with Semi-Supervised Learning Strategy. Adv. Mater..

[52] Popova M., Isayev O., Tropsha A. (2018). Deep reinforcement learning for de novo drug design. Sci. Adv..

[53] Mishra M. (2018). Encyclopedia of Polymer Applications, 3 Volume Set.

[54] Kumar J.N., Li Q., Jun Y. (2019). Challenges and opportunities of polymer design with machine learning and high throughput experimentation. MRS Commun..

[55] Kumar J.N., Li Q., Tang K.Y.T., Buonassisi T., Gonzalez-Oyarce A.L., Ye J. (2019). Machine learning enables polymer cloud-point engineering via inverse design. npj Comput. Mater..

[56] Nápoles G., Grau I., Bello R. (2012). Constricted Particle Swarm Optimization based algorithm for global optimization. Polibits.

[57] Khadilkar M.R., Paradiso S., Delaney K.T., Fredrickson G.H. (2017). Inverse Design of Bulk Morphologies in Multiblock Polymers Using Particle Swarm Optimization. Macromolecules.

[58] Hiraide K., Hirayama K., Endo K., Muramatsu M. (2021). Application of deep learning to inverse design of phase separation structure in polymer alloy. Comput. Mater. Sci..

[59] Ramprasad R., Batra R., Pilania G., Mannodi-Kanakkithodi A., Kim C. (2017). Machine learning in materials informatics: Recent applications and prospects. npj Comput. Mater..

[60] Zhu M.X., Deng T., Dong L., Chen J.M., Dang Z.M. (2021). Review of machine learning-driven design of polymer-based dielectrics. IET Nanodielectr..

[61] Mannodi-Kanakkithodi A., Pilania G., Huan T.D., Lookman T., Ramprasad R. (2016). Machine Learning Strategy for Accelerated Design of Polymer Dielectrics. Sci. Rep..

[62] Wu S., Lambard G., Liu C., Yamada H., Yoshida R. (2020). iQSPR in XenonPy: A Bayesian Molecular Design Algorithm. Mol. Inf..

[63] Gurnani R., Kamal D., Tran H., Sahu H., Scharm K., Ashraf U., Ramprasad R. (2021). polyG2G: A Novel Machine Learning Algorithm Applied to the Generative Design of Polymer Dielectrics. Chem. Mater..

[64] Li C., Li Q., Kaneti Y.V., Hou D., Yamauchi Y., Mai Y. (2020). Self-assembly of block copolymers towards mesoporous materials for energy storage and conversion systems. Chem. Soc. Rev..

[65] Liu Z., Zhu D., Raju L., Cai W. (2021). Tackling Photonic Inverse Design with Machine Learning. Adv. Sci..

[66] Bendsoe M.P., Sigmund O. (2003). Topology Optimization: Theory, Methods, and Applications.

[67] Molesky S., Lin Z., Piggott A.Y., Jin W., Vucković J., Rodriguez A.W. (2018). Inverse design in nanophotonics. Nat. Photonics.

[68] Angeris G., Vučković J., Boyd S.P. (2019). Computational Bounds for Photonic Design. ACS Photonics.

[69] Liu D., Tan Y., Khoram E., Yu Z. (2018). Training Deep Neural Networks for the Inverse Design of Nanophotonic Structures. ACS Photonics.

[70] Qu Y., Zhu H., Shen Y., Zhang J., Tao C., Ghosh P., Qiu M. (2020). Inverse design of an integrated-nanophotonics optical neural network. Sci. Bull..

[71] Jensen J.S., Sigmund O. (2011). Topology optimization for nano-photonics. Laser Photonics Rev..

[72] Liu J., Gaynor A.T., Chen S., Kang Z., Suresh K., Takezawa A., Li L., Kato J., Tang J., Wang C.C.L. (2018). Current and future trends in topology optimization for additive manufacturing. Struct. Multidiscip. Optim..

[73] Long Y., Ren J., Li Y., Chen H. (2019). Inverse design of photonic topological state via machine learning. Appl. Phys. Lett..

[74] Pilozzi L., Farrelly F.A., Marcucci G., Conti C. (2018). Machine learning inverse problem for topological photonics. Commun. Phys..

[75] Jiang J., Sell D., Hoyer S., Hickey J., Yang J., Fan J.A. (2019). Free-form diffractive metagrating design based on generative adversarial networks. ACS Nano.

[76] Liu Z., Zhu Z., Cai W. (2020). Topological encoding method for data-driven photonics inverse design. Opt. Express.

[77] Kudyshev Z.A., Kildishev A.V., Shalaev V.M., Boltasseva A. (2020). Machine learning–assisted global optimization of photonic devices. Nanophotonics.

[78] Braham E.J., Davidson R.D., Al-Hashimi M., Arroyave R., Banerjee S. (2020). Navigating the design space of inorganic materials synthesis using statistical methods and machine learning. Dalton Trans..

[79] Noh J., Gu G.H., Kim S., Jung Y. (2020). Machine-enabled inverse design of inorganic solid materials: Promises and challenges. Chem. Sci..

[80] Kim S., Noh J., Gu G.H., Aspuru-Guzik A., Jung Y. (2020). Generative Adversarial Networks for Crystal Structure Prediction. ACS Cent Sci..

[81] Dan Y., Zhao Y., Li X., Li S., Hu M., Hu J. (2020). Generative adversarial networks (GAN) based efficient sampling of chemical composition space for inverse design of inorganic materials. npj Comput. Mater..

[82] Rosales A.R., Wahlers J., Limé E., Meadows R.E., Leslie K.W., Savin R., Bell F., Hansen E., Helquist P., Munday R.H. (2018). Rapid virtual screening of enantioselective catalysts using CatVS. Nat. Catal..

[83] Qin J., Chen Q., Yang C., Huang Y. (2016). Research process on property and application of metal porous materials. J. Alloys Compd..

[84] Ferey G. (2001). Materials science. The simplicity of complexity–rational design of giant pores. Science.

[85] Kim B., Lee S., Kim J. (2020). Inverse design of porous materials using artificial neural networks. Sci. Adv..

[86] Yao Z., Sánchez-Lengeling B., Bobbitt N.S., Bucior B.J., Kumar S.G.H., Collins S.P., Burns T., Woo T.K., Farha O.K., Snurr R.Q. (2021). Inverse design of nanoporous crystalline reticular materials with deep generative models. Nat. Mach. Intell..

[87] Wan J., Jiang J.-W., Park H.S. (2020). Machine learning-based design of porous graphene with low thermal conductivity. Carbon.

[88] Wang T., Zhang C., Snoussi H., Zhang G. (2019). Machine Learning Approaches for Thermoelectric Materials Research. Adv. Funct. Mater..

[89] Recatala-Gomez J., Suwardi A., Nandhakumar I., Abutaha A., Hippalgaonkar K. (2020). Toward Accelerated Thermoelectric Materials and Process Discovery. ACS Appl. Energy Mater..

[90] Zheng B., Yang J., Liang B., Cheng J.-C. (2020). Inverse design of acoustic metamaterials based on machine learning using a Gauss–Bayesian model. J. Appl. Phys..

[91] Ismail M.S., Moghavvemi M., Mahlia T.M.I. (2013). Characterization of PV panel and global optimization of its model parameters using genetic algorithm. Energy Convers. Manag..

[92] Jadrich R.B., Lindquist B.A., Truskett T.M. (2017). Probabilistic inverse design for self-assembling materials. J. Chem. Phys..

[93] Forte A.E., Hanakata P.Z., Jin L., Zari E., Zareei A., Fernandes M.C., Sumner L., Alvarez J., Bertoldi K. (2022). Inverse Design of Inflatable Soft Membranes Through Machine Learning. Adv. Funct. Mater..

[94] Lininger A., Hinczewski M., Strangi G. (2021). General Inverse Design of Layered Thin-Film Materials with Convolutional Neural Networks. ACS Photonics.

[95] Jiang R., Da Y., Han X., Chen Y., Deng Y., Hu W. (2021). Ultrafast Synthesis for Functional Nanomaterials. Cell Rep. Phys. Sci..

[96] Dou S., Xu J., Cui X., Liu W., Zhang Z., Deng Y., Hu W., Chen Y. (2020). High-Temperature Shock Enabled Nanomanufacturing for Energy-Related Applications. Adv. Energy Mater..

[97] Genty G., Salmela L., Dudley J.M., Brunner D., Kokhanovskiy A., Kobtsev S., Turitsyn S.K. (2021). Machine learning and applications in ultrafast photonics. Nat. Photonics.

[98] Kitchin J.R. (2018). Machine learning in catalysis. Nat. Catal..

